# Faulty risk-of-bias assessment in a meta-analysis of hydroxyethyl starch for non-septic ICU patients: a rebuttal

**DOI:** 10.1186/s13054-015-1168-2

**Published:** 2015-12-24

**Authors:** Michael James, Ivan Joubert, William Lance Michell, Andrew Nicol, Pradeep Navsaria, Rencia Gillespie

**Affiliations:** Department of Anaesthesia, University of Cape Town and Groote Schuur Hospital, Anzio Road, Cape Town, Western Cape 7925 South Africa; Division of Critical Care and Department of Anaesthesia, University of Cape Town and Groote Schuur Hospital, Cape Town, Western Cape South Africa; Division of Critical Care and Department of Surgery, University of Cape Town and Groote Schuur Hospital, Anzio Road, Cape Town, Western Cape 7925 South Africa; Trauma Unit, Division of General Surgery, University of Cape Town and Groote Schuur Hospital, Anzio Road, Cape Town, Western Cape 7925 South Africa

The Fluids in Resuscitation of Severe Trauma (FIRST) study meets all of the criteria for assessment as a low risk of bias study, contrary to the unsupported allegations by Bayer and Reinhardt.

We dispute the letter from Bayer and Reinhart together with the response from He et al. [1]. Bayer and Reinhart claim that the FIRST study [2] has a high risk of bias and cite two non-peer-reviewed letters from themselves and Finfer to support this claim. However, these authors fail to cite the extensive responses that more than adequately cover their queries [3].

Bayer and Reinhart claim that there was selective outcome reporting, but all of the outcomes listed in the methods of the FIRST trial have been reported. As with all published work, space constraints imposed by the journal limit the amount of detail that can be included. In our paper all statistically significant results were reported in detail and other outcomes that were not significant were only reported briefly as is the norm. These non-significant outcomes were more than adequately addressed in the subsequent correspondence. There is therefore no basis for the claim that this study shows a high risk of bias. Indeed, in the initial letter from Bayer and Reinhart, their own bias is clearly illustrated in their attempts to draw inferences from non-significant data.

In our view, the FIRST study meets all of the criteria for assessment as a low risk of bias study and we dispute the concession made by He et al. [4] regarding the risk of bias of this study. Our view is that the original analysis in the published paper reflects the correct scientific position and that the modified Jadad score of 6 allocated to this study is appropriate.

## Abbreviation

FIRST: Fluids in Resuscitation of Severe Trauma
